# Edible Bird's Nest as a Potential Cognitive Enhancer

**DOI:** 10.3389/fneur.2022.865671

**Published:** 2022-05-06

**Authors:** Su-Peng Loh, Shi-Hui Cheng, Wael Mohamed

**Affiliations:** ^1^Department of Nutrition, Faculty of Medicine and Health Sciences, Universiti Putra Malaysia, Serdang, Malaysia; ^2^Faculty of Science and Engineering, School of Biosciences, University of Nottingham Malaysia, Semenyih, Malaysia; ^3^Clinical Pharmacology Department, Menoufia Medical School, Menoufia University, Shebin El Kom, Egypt; ^4^Basic Medical Science Department, Kulliyyah of Medicine, International Islamic University Malaysia (IIUM), Selayang, Malaysia

**Keywords:** cognitive enhancers, edible bird's nest, dietary, neuroprotection, dementia

## Abstract

Cognitive enhancement is defined as the augmentation of the mind's core capabilities through the improvement of internal or external information processing systems. Recently, the focus has shifted to the potential therapeutic effects of natural products in improving cognitive function. Edible bird's nest (EBN) is a natural food substance derived from the saliva of swiftlets. Until today, EBN is regarded as a high-priced nutritious food with therapeutic effects. The effectiveness of dietary EBN supplementation to enhance brain development in mammals has been documented. Although the neuroprotection of EBN has been previously reported, however, the impact of EBN on learning and memory control and its potential as a cognitive enhancer drug remains unknown. Thus, this article aims to address the neuroprotective benefits of EBN and its potential effect as a cognitive enhancer. Notably, the current challenges and the future study direction in EBN have been demonstrated.

## Introduction

Cognitive enhancers (CEs), also known as nootropics, are supplements consumed to improve memory, enhance concentration, and boost energy levels and alertness. Attempts to improve cognitive function and memory have become a study hotspot recently. CE is also being tested in Alzheimer's disease (AD) and in aging study, with a focus to reverse the cognitive impairment associated with dementia (1, 2). Consistent with this, the so-called CE drugs are reported to be used widely in clinical practice. For instance, acetylcholinesterase inhibitors (AChEIs) and memantine are now conventional therapies for neurodegenerative illnesses such as AD and Parkinson's disease (3–5). While treating cognitive dysfunction symptoms in neurodegenerative disorders in patients using CE drug may be beneficial, the prospect of significantly improving cognitive memory and learning in otherwise healthy individuals often raise ethical issues (6). Moreover, to the best of our knowledge, none of the drugs can reverse the damage of the neurons that subsequently leads to neurodegenerative diseases.

Reactive oxygen species (ROS), which are the sources of oxidative stress, are especially active in the brain and neuronal tissue. Glial cells and neurons are more vulnerable to free radicals, especially ROS, and this ultimately leads to neuronal damage. Furthermore, the brain is highly exposed to oxidative stress as the brain cells require a large amount of oxygen. Free radical overproduction can induce oxidative stress to biomolecules (DNA, lipids, and protein), which can lead to many chronic diseases, including neurodegenerative diseases (7). A number of evidence indicates that several interrelated cerebral pathways such as oxidative stress, neuroinflammation, and altered gene expression could result in the death of neuronal cells (8, 9). Recently, attention has turned to the potential therapeutic effects of natural products to improve cognitive performance (Table 1). Food-based antioxidants and herbal have then become increasingly popular after an association between neuroprotective function and diet has been reported (32). New study on edible bird's nest (EBN) has suggested its neuroprotective effects against AD, with studies reporting its ability to suppress neuroinflammation and neuronal cell death (33–35). Thus, EBN is believed to have a favorable effect on cognitive function. Previous researchers focused on the neuroprotective effect of EBN (33–35); however, no light has been shed on the effect of EBN as a CE; therefore, it is worthwhile to review EBN and its potential as CE. In addition, we have discussed the recent problem confronting researchers in the field of EBN study and addressed the future study prospects of EBN, including the development of EBN as a potential supplement of neurodegenerative diseases.

**Table 1:** Summary of some promising cognition-enhancing natural substances.

**Name**	**CNS benefits**	**Mechanism of action**	**Literature's support**
Bacopa monniera (*Water yssop, Brahmi*)	Facilitation of memory retention. Alleviation of the symptoms of anxiety and convulsive disorders. Powerful antioxidant actions.	Although the plant contains a variety of biologically active ingredients, including alkaloids, saponins, and sterols, the triterpenoid saponins known as “bacosides” are responsible for the memory-enhancing properties.	• Bacopa has been shown to have antioxidant qualities (10) and to protect mental function in epileptic patients taking the medication phenytion (11). • In rats, bacopa treatment enhanced learning (12) and exhibited antidepressant action (13). • Bacopa enhanced learning and memory in healthy humans, with the greatest benefit occurring after 12 weeks (14).
Fava beans (*Viciafaba*)	The L-dopa found in Fava beans promotes memory, energy, a sense of wellbeing, and sexual drive, as well as aids in the treatment of Parkinson's Disease symptoms.	Fava beans has high concentrations of L-dopa, an amino acid that is enzymatically converted to the neurotransmitter dopamine.	• For 12 weeks, Parkinson's disease patients were given fava bean seed powder, while a control group received synthetic therapies. According to the Unified Parkinson's Disease Rating Scale, Fava Beans were beneficial with few unwanted effects (15, 16).
Ashwagandha (*Withania somnifera*)	Ashwagandha is said to possess anti-inflammatory, anticancer, anti-stress, antioxidant, cognitive enhancing, and rejuvenating qualities.	Ashwagandha contains flavonoids and withanolide-class active compounds. Ashwagandha exhibits GABA-like activity (anxiolityc activity) and has been shown to raise the brain's acetylcholine level (cognition enhancement).	• According to study, ashwagandha increases the growth of axons and dendrites and the quantity of antioxidants in the brain (17). • Numerous mouse studies demonstrated that Ashwagandha exhibited memory-enhancing, anti-anxiety, antidepressant, and testosterone-like properties (18–20).
Celastrus paniculatus (CP) (*Jyotishmati*)	Cognitive enhancing effect and powerful antioxidant activities are attributed to extracts of CP	Although some evidence suggests that the active compounds in CP (Bacosides A and B) reduce the activity of norepinephrine, dopamine, and serotonin, the mechanism by which CP exerts its cognitive effect is not completely understood.	• Chronic administration of seeds of CP to rats selectively corrected the deficit in spatial memory caused by scopolamine (21). • Additionally, significant antioxidant activity was demonstrated in the brains of rats (22).
Origanum majorana	Acetylcholinesterase (AChE) inhibitors, both natural and synthetic, increase the cholinergic tone in the brain and are beneficial in Alzheimer's disease and cognitive enhancement.	Ursolic acid, one of the active ingredients of origanum majorana (OM) and other Labiatae family herbs, is a highly effective natural AChE inhibitor (23).	• Ursolic acid demonstrated antioxidant action, reducing Abeta neurotoxicity in rat's brain (24).
Alpha lipoic acid (ALPA, thioctic acid)	Enhances memory by healing oxidative stress-induced cell damage and protects neurons from oxygen deprivation and poor blood flow.	Increases glutathione, vitamin C, and E levels and reduces reactive oxidant concentrations. Additionally, it decreases glucose levels and protects the mitochondria (25).	• Double blind placebo-controlled trials demonstrate the antioxidant efficacy in the treatment of several types of poisoning (26), aging (27), and diabetes (28). • ALPA was able to cure memory impairment and oxidative stress in aged mice in animal tests (29, 30) or in combination with acetyl-L-camitine (31).

## Overview of Edible Bird's Nest

Edible bird's nest is a salivary secretion created by swiftlets. There are three main types of swiftlets genera known to produce EBN: *Collocalia, Aerodramus*, and *Hydrochous* (36). During the nesting and breeding season, the sublingual gland of swiftlets increases in weight and reaches their maximum secretory activity (37, 38). Swiftlets, the insectivorous birds, build its nest with secretions from their specialized salivary glands. Only ~70–90% of the nest involves mucus production, with feathers and nest-feeding insects rounding out the nest composition (36, 37). The nests are built over a duration of 35 days. The nests are graded according to the number of feathers, size, color, and impurity *via* the physical appearance. The growth and reproduction of swiftlets required specific environmental conditions, including humidity of about 90% and temperature 28–30°C (39). Therefore, swiftlets are only found in areas with a suitable condition in Southeast Asian countries, including Thailand, Malaysia, Vietnam, and the Philippines.

More than 24 different swiftlet species create nests for their young all around the world, but only a handful of them are edible. Both the *Aerodramus fuciphagus* and *Aerodramus maximus* lay white and black nests, respectively, and are the most exploited and recognized swiftlet species in Malaysia (38). Red nests or blood nests, also known as *Xueyan* in Chinese, are occasionally found in the caves and swiftlet houses. It is believed that red nests are of an excellent quality (40) and believed to have increased health benefits and, therefore, fetch a relatively higher price than white nests in the market (41). However, both the white and red EBNs showed relatively similar amino acid levels, which were 63 and 62%, respectively (38). The reddening of EBN has been reported to be associated with the emission of bird soil in hot and humid environments or a chemical reaction involving sodium nitrite dissolved in 2% hydrochloric acid, but the mechanism is unclear (42). Two researchers have previously provided conflicting statements about the red color of EBN. According to Wong et al. (43), red EBN is formed due to the oxidation of Fe irons in acidic mammalian chitinase (AMCase)-like proteins, whereas Shim and Lee (44) hypothesized that the red color is caused by a xanthoproteic reaction. Nevertheless, the color of EBN in Malaysia usually ranges from pale to yellowish; this could be attributed to its minerals, phenolic content, nitrite, and environmental factors (42).

Since the Tang Dynasty (681 AD), the Chinese community has recognized EBN as a precious food and medicine known as “Caviar of the East,” a title it has held since (38). In ancient times, the EBN soup was created by double boiling with rock sugar and was only available to the emperor and the affluent. It has been used by the Chinese for more than a 1,000 years for its nutritional content and health benefits, despite its reputation as a pricey traditional medicine. Owing to its esteem as a delicacy food in traditional Chinese medicine, EBN will continue to be considered a healthy food and beauty enhancer that can treat various respiratory and digestive system ailments. In addition, it boosts the immune system and enhances the appearance of aging skin. Asthma, cough, and stomach ulcers have also been shown to benefit from EBN (38, 45–47). EBN has recently been shown to have antiviral and neuroprotective properties by suppressing influenza infection (48–50). EBN has antioxidant, anti-inflammatory, and bone-strengthening properties (51, 52). Due to EBNs medicinal and delectable qualities, it has become more widely known worldwide (47). EBN has been reported worldwide as a major element in health-supplementing foods, beverages, and beauty enhancers (46).

## Active Compounds of Edible Bird's Nest

Edible bird's nest has a distinct composition and its consumption may promote human health (38, 53). Proteins and carbohydrates are two of the most biologically active components of EBN and they are crucial while determining the drug's effectiveness. The protein makes up the highest composition in EBN, which is about 50–60% of EBNs weight on average. Amino acids, the building blocks of proteins, are necessary for the body's cells to develop and regenerate and for the formation of brain neurotransmitters, antibodies, and immunoglobulin (38, 54, 55). The essential amino acid found in EBN (17.8g/100 g) was far greater than in other protein-rich foods such as egg (4.7–7.0 g/100 g) and milk (1.1 g/100 g) (53). Out of the 20 types of amino acids needed by humans, 18 amino acids are detected in EBN, including 9 essential amino acids (phenylalanine, valine, threonine, histidine, tryptophan, isoleucine, methionine, lysine, and leucine) (56). In addition, two of the essential amino acids found in EBN, namely, lysine and tryptophan, are not present in most plant proteins, suggesting that EBN could provide a complete amino acid for vegetarians. Nonetheless, the total amino acids are different based on various geographical locations. The varying composition of EBN amino acids is mostly attributable to the diverse collection sites and cave or man-made housing types used by EBN (38, 57). Human health greatly benefits from the EBNs protein and carbohydrate composition (38, 55, 58–60). In 2017, a study found that EBNs hallmark peptide is a mucin-like protein, which is used to classify EBN based on its color and collecting locations (61). EBNs protein content rises because of its digestion in the stomach and by its acidic enzymes (43).

Researchers found that EBN contains a high concentration of serine, threonine, and aspartic acids, glutamic acids, prolines, and valines (58, 59). Glycoproteins (lactoferrin and ovotransferrin) are the molecules that provide EBN with its special usefulness and are reported to contribute to the neuroprotective activity (38, 43, 47, 54, 59, 61). An important component of white EBN is the aromatic amino acid tyrosine, which has antidepressant and analgesic properties (38).

Edible bird's nests second most important component is carbohydrates, including N-acetylneuraminic acid (sialic acid), galactosamine, N-acetylglucosamine, and N-acetylgalactosamine. The main carbohydrate present in EBN is sialic acid, with a content of about 10%. EBNs sialic acid, which has pharmacological effects on human health, is the only indicator that allows the grading of diverse EBN (53). Sialic acid is contained in EBN in the form of N-acetylneuraminic acid (Neu5Ac) (Neu5Ac or NANA) (62–65). It is important to note that sialic acid facilitates neuronal outgrowth, synaptic transmission, and brain development. Increasing the activity of brain cells and improving cognitive abilities are both helped by a diet high in sialic acid (55, 66, 67). Compared to foods high in sialic acid, such as human milk and chicken egg yolk, EBN has a higher concentration from 7.2 to 13.6 g/100 g (53). Due to its high content, EBN has a positive effect on brain development, flu prevention, immune augmentation, cell proliferation, and neurological improvement (43, 50, 66, 68, 69).

It is also worth noting that the fat content in EBN is <0.5%, showing that EBN is a low-fat food. In particular, the triglyceride of EBN is rich in polyunsaturated fatty acids (48%) (70). In summary, EBN is considered a complete food rich in proteins and carbohydrates. Key nutrients, including essential amino acids and sialic acids, may have great health benefits in humans. However, to this day, the role of EBN and cognitive function is not thoroughly researched.

## Antioxidant Effects of Edible Bird's Nest

The human body is equipped with several antioxidant systems that safeguard it from the oxidative damage induced by normal metabolic activity (71). Antioxidants in the meal are capable of fighting cell-disrupting effects. These antioxidants act either independently or in concert with endogenous processes. While it has been proven that the antioxidant effects of food are advantageous to human health, their absence may induce a range of illnesses caused by excessive oxidative stress. Many fruits and vegetables have been shown to have anticancer and anti-inflammatory effects. Thus, people who regularly consume antioxidant-rich fruits and vegetables reduce their chance of acquiring illnesses caused by free radicals (58). Antioxidants have received significant attention in the modern period due to their ability to treat oxidative stress-related diseases.

Edible bird's nests antioxidant properties are attributed to the inclusion of several bioactive components, including amino acids, sialic acid, triacylglycerol, vitamins, lactoferrin, fatty acids, minerals, and glucosamine (70, 72, 73). Due to the inclusion of two key components, ovotransferrin and lactoferrin, EBN displayed antioxidative action (74). In addition, the researchers proved their capacity to protect human neuroblastoma SH-SY5Y (HNS) cells against the toxicity caused by hydrogen peroxide (H_2_O_2_). Furthermore, lactoferrin, ovotransferrin, and EBN altered the transcription of antioxidant-related genes linked with neuroprotection (74). Yida et al. (52) assessed the bioavailability and antioxidant activity of EBN water extracts *in vitro* using the oxygen radical absorbance capacity (ORAC) and 2,2′-azino-bis-(3-ethylbenzothiazoline-6-sulfonic acid) (ABTS) methods. In all the ABTS and ORAC studies, the undigested EBN water extract displayed low antioxidant activity (about 1% at 1,000 g/ml). On the other hand, EBN samples digested with pepsin, pancreatin, and bile extract at comparable concentrations revealed an ~38 and 50% increase in antioxidant activity in the ABTS and ORAC assays, respectively. In addition, it was shown that the EBN extracts were non-toxic to human hepatocellular carcinoma (HEPG2) cells and protected them against H_2_O_2_-induced toxicity.

After gastrointestinal digestion, the antioxidant effect of EBN will be enhanced (52, 75). Study carried out by Yida et al. (64) reported that EBN has the ability to reduce the risk of hypercoagulation associated with cardiovascular disease (CVD). Results showed that the EBN-treated group can improve the lipid profile and lower the blood sugar level and total cholesterol by reducing oxidative stress compared to the control group. In 2015, the same study group demonstrated the effect of EBN on a high-fat diet (HFD) induced oxidative stress in a rat model (76). The results showed that EBN could reduce the oxidative stress and inflammation triggered by HFD *via* transcriptional control of hepatic antioxidant gene expression related to inflammation. The results support the effectiveness of EBN in the prevention of inflammation and oxidative stress induced by obesity.

In addition, a study done by Ghassem et al. suggested that protein hydrolysate of EBN possesses antioxidant properties and can scavenge the free radical (72). A similar study was reported on improving the level of superoxide dismutase (SOD), estrogen, malondialdehyde, and lipid profile of the ovariectomized rats with 12 weeks of EBN supplementation in the diet (77). These findings highlight the value of EBN to prevent cardiometabolic disease induced by estrogen deficiency. Hu et al. studied the antiaging effect of EBN in the *Drosophila melanogaster* model. The study showed that EBN could decrease mortality rates and lipid peroxidation *via* increasing the antioxidant enzyme activity (78). Likewise, study carried out by Albishtue et al. (79) to evaluate the effect of EBN supplementation on uterine function and embryo implantation rate has proven that EBN enhances the antioxidative activity and decreases oxidative stress level, which enhances embryo implantation (79).

Edible bird's nest can significantly enhance memory on hippocampal neurons (SH-SY5Y neuroblastoma cells) by inhibiting oxidative stress (75). In addition, EBN contains glycoproteins such as lactoferrin (LF) and ovotransferrin (OVF), which were reported to have neuroprotective activity and antioxidant through scavenging free radical species in SH-SY5Y cells (80, 81). When EBN was tested on SH-SY5Y cells, Hou et al. found that the antioxidant and protective effects on the cells were also due to its components LF and OVF (77). These findings showed that EBN has antiaging and antineurodegenerative properties. It was also discovered that the EBN extract protects dopaminergic neurons from 6-hydroxydopamine-induced degeneration (75). These findings indicated that EBN might confer a potential therapeutic for neurodegenerative disorders such as AD and Parkinsonism exacerbated by oxidative stress.

In summary, the augmentation of EBNs antioxidant activity after digestion revealed its post-consumption functional benefits. However, additional study, such as *in-vivo* investigations, is necessary to fully evaluate the clinical importance of EBN.

## Neuroprotective Effects of Edible Bird's Nest

Over the last several years, many experts have undertaken studies on EBN and its neuroprotective qualities. For example, Yew et al. investigated the neuroprotective properties of EBN extracts in HNS cells (75). The study indicated that pancreatin-digested EBN extract significantly decreased cell mortality in HNS cells at concentrations up to 75 g/ml, although the highest non-toxic dosage of EBN water extract was twice that much (150 g/ml). EBN inhibits apoptosis induced by 6-hydroxydopamine (6HD) in HNS cells, as determined by nuclear staining and morphological inspection. Notably, when the EBN extract was digested, cell viability was dramatically enhanced compared to the EBN water extract. Nonetheless, EBN water extract was shown to have important activities in preventing caspase-3 cleavage, controlling the early apoptotic effect on the phosphatidylserine externalization membrane and neuron recovery in the presence of ROS. Thus, EBN may be a viable nutraceutical option for preventing neurodegenerative diseases related to oxidative stress. In a second study, Hou et al. (74) revealed the impact of EBN on the toxicity depletion of H_2_O_2_ in HNS cells. Lactoferrin and ovotransferrin were reported to protect against H_2_O_2_-induced toxicity and cytotoxicity when incorporated in EBN. The contents of EBN further reduced ROS by enhancing the scavenging process, which is consistent with a subsequent study (33), which discovered that supplementing with EBN inhibited the production of oxidative markers ROS and thiobarbituric acid reactive substances (TBARS) in a Wistar rat model of Lipopolysaccharide (LPS)-induced neuroinflammation. EBN may act as a neuroprotective agent against oxidative stress and H_2_O_2_-induced cytotoxicity in cells, based on these findings.

Although various investigations on the neuroprotective benefits of EBN have been undertaken (Table 2), present scientific knowledge is unable to determine which EBN components or combinations thereof display neuroprotective capabilities. As a result, further study on EBN is warranted in the near future to address this gap.

**Table 2:** Summary of studied effects of edible bird's nest (EBN) extract related to cognition.

**Effects**	**Sample preparation**	**Experimental model**	**Dosage**	**Results**	**Proposed cognitive mechanism**	**References**
Antioxidant effect	Water extraction	Human neuroblastoma cell (SH-SY5Y)	1,000 μg/ml	EBN demonstrated protective effects against hydrogen peroxide-induced toxicity and cell oxidative stress on SH-SY5Y cells. Lactoferrin and ovotransferrin also possess antioxidant capacities on SH-SY5Y cells	• EBN and its ingredients reduced hydrogen peroxide-induced cytotoxicity, and decreased ROS by increasing scavenging activity. • Suggested active ingredient: Lactoferrin and ovotransferrin	(74)
	Water extraction	Human hepatocellular liver carcinoma (HepG_2_) cells	1,000 μg/mL	EBN extracts were non-toxic to HEPG_2_ cells, and EBN extracts protected HEPG_2_ cells from hydrogen peroxide induced-toxicity.	• EBN and its ingredients reduced hydrogen peroxide-induced cytotoxicity, and digested EBN samples had significantly enhanced antioxidant activities	(52)
	Powder	Rat model-high fat diet (HFD)was fed to rats for 12 weeks and compared with HFD + Simvastatin and HFD + EBN (2.5 or 20 %)	EBN (2.5 or 20 %)	C-reactive protein (CRP), interleukin (IL) 6 and tumor necrosis factor alpha (Tnf-α) were decreased in the EBN and Simvastatin groups.	• EBN reduced oxidative stress and inflammation due to high fat diet via modulating hepatic antioxidant and inflammation-related genes, and its results were superior than Simvastatin. Suggested active ingredient: sialic acid, lactoferrin and ovotransferrin	(76)
	Water extraction	HepG2 cells and human normal lung (MRC-5) cells	800 μg/mL	• EBN peptides showed a protective effect on H_2_O_2_-induced HepG2 cells by increasing cell viabilities up to 91.44 and 92.31%, respectively. • EBN peptides showed no *in vitro* cytotoxicity on human lung MRC-5 cells	• The protective effect of EBN is mainly caused by peptides' scavenging ability of H2O2 radicals • Suggested active ingredient: Pro-Phe-His-Pro-Tyr and Leu-Leu-Gly-Asp-Pro	(72)
	Powder	*Drosophila melanogaster*	food medium supplemented with 0 g/kg (control), 1, 3, and 9 g/kg of EBN	Results of ferric reducing antioxidant power (FRAP) showed that the EBN-treated groups had higher total antioxidant activity in a dose-dependent manner as compared to control	• EBN enhanced the antioxidant capacity by increasing the SOD and catalase activities (CAT) whereas the MDA level was decreased. • Suggested active ingredient: sialic acid	(78)
	Powder	Rat	30, 60, and 120 mg/kg body weight of EBN	EBN supplemented groups had normal uterine structures and higher morphological parameters of uteri than the control, despite exposure to lead acetate toxicity. SOD levels were also increased in EBN supplemented groups	• EBN reduce the effects of LA toxicity on the uterus by increasing enzymatic antioxidant (SOD) activity as well as expressions of cell proliferation factors such as epidermal growth factor (EGF), vascular endothelial growth factor (VEGF), and proliferating cell nuclear antigen (PCNA). • Suggested active ingredient: sialic acid, EGF-like compound	(79)
Neuroprotective effect	Water extraction	Human neuroblastoma cell (SH-SY5Y)	0 to 500 μg/ml	EBN treatment lowers the level of 6- hydroxydopamine- induced apoptosis in SH-SY5Y cells, as evidenced by morphological and nuclear staining data	• EBN treatment reduces the level of 6- hydroxydopamine- induced apoptotic changes in SH-SY5Y cells that was revealed by morphological and nuclear staining observations	(75)
	Water extraction	Human neuroblastoma cell (SH-SY5Y)	1,000 μg/ml	EBN and its content (lactoferrin and ovotransferrin) enhanced scavenging activity by reducing H_2_O_2_- induced cytotoxicity and decreasing radical oxygen species	• EBN acts as a neuroprotective (SH- SY5Y human neuroblastoma cell) agent against H_2_O_2_- induced cytotoxicity and cell oxidative stress • Suggested active ingredient: Lactoferrin and ovotransferrin	(74)
	Powder	Ovariectomized rats	1.2, 0.6, and 0.3 g/kg body weight	EBN and estrogen enhanced spatial learning and memory and increased serum estrogen and hippocampal sirtuin-1 (SIRT1) expression, and EBN groups did not show as much toxicity to the liver as the estrogen group	• The effect of EBN in improving cognition and memory in ovariectomized rats is probably mediated by enhancing the SIRT1-mediated neuronal plasticity	(69)
	Alcohol extraction	2VO rat model	60 mg/kg, 120 mg/kg	EBN demonstrated neuroprotective effects especially for Hippocampus CA1 area	• They underscored the neuroprotective effects of EBN with brain ischemia in rat animal model	(34)
	Powder	Wistar rats	125 mg/kg, 250 mg/kg, and 500 mg/kg	Treatment with EBN inhibited the LPS-induced upregulation of proinflammatory cytokines (TNF-α, IL-1β, and IL-6) and oxidative markers (ROS and TBARS) in the hippocampus.	• Anti-inflammatory effect by EBN may be attributed to sialic acid content	(33)
	Powder	ICR mice	9 g dry EBN in every 182 g water	administration with EBN in maternal mice during pregnancy or lactation period can improve the learning and memory functions in their offspring	• Maternal EBN administration improved the spatial learning in the offspring by increasing SOD and choline acetyltransferase (ChAT) activity and, decreasing the levels of MDA and activities of acetylcholinesterase (AChE). Moreover, BDNF levels for CA1, CA2, and CA3 regions in hippocampi and the numbers of dyed neurons in CA1, CA2, CA3, and DG regions among the offspring were significantly enhanced.	(50)
	Water extraction	CJ57B/6 breeder's mice	10 mg/kg body weight	Dietary EBN supplementation showed an upregulation of GNE, ST8SiaIV, SLC17A5, and BDNF mRNA associated with an improvement in Y-maze cognitive performance in both generations of the animal.	• EBN supplementation improved the learning and memory function of F1 and F2 mice in the Y-maze test by increasing brain gene expression and synaptic vesicle densities	(82)
	Water extraction	BALB/c mice	20, 40, 60, 80, and 100 μg/ml sialic acid	Sialic acid extract and sialic acid standard at 60 μg/mL (0.6 ppm) increased cell viability and showed no cytotoxicity effects in pheochromocytoma and neuroblastoma cell lines. An administration of higher dose of sialic acid at 0.6 ppm in animals improved Y-maze test performance	• The presence of epidermal growth factor-like activity in sialic acid extract from EBN play a key role in growth-stimulating normal cellular processes such as proliferation, differentiation, and development.	(66)
	Powder	Ovariectomized rats	6, 3, and 1.5% EBN	EBNs significantly decreased estrogen deficiency-associated serum elevation of advanced glycation end-products (AGEs), and they changed redox status as evidenced by oxidative damage (MDA content) and enzymatic antioxidant defense (SOD and catalase) markers	• EBN has potential for neuroprotection against estrogen deficiency-associated senescence, by decreasing serum AGEs, reducing hippocampal and frontal cortical caspase 3 protein and MDA levels, and balancing activities of anti-oxidant enzymes in the hippocampus and frontal cortex of ovariectomized female rats	(83)

## Edible Bird's Nest Effects on Cognition

The newborn infant's growth and development require excellent demands on the nutrition supply, especially in the brain. Any food deficit has a profound effect on the development of the brain. One of the important nutrients in EBN is sialic acid and, interestingly, it has been shown to improve brain function. Studies have revealed that sialic acid can improve a child's intelligence and brain functioning by enhancing the synaptic route and ganglioside distribution (67, 84). Upregulation of several genes in the physiological system associated with cognitive development occurs when sialic acid is used as a dietary supplement (85).

Brain-derived neurotrophic factor (BDNF) is a key molecule involved in learning and memory, particularly important for memory processes such as the hippocampus and parahippocampal areas (86). When EBN was administered to pregnant and lactating women, it increased BDNF and sialic acid levels in the hippocampus, according to study conducted by Xie et al. (50). The hippocampus CA1, CA2, and CA3 regions see an increase in neuronal cell density when EBN is administered. EBN increased the offspring learning and memory abilities by increasing superoxide dismutase (SOD) and choline acetyltransferase (ChAT) activities, but lowered its acetylcholinesterase (AChE) activity (50). Similar results were observed in the effect of EBN on mice. The *BDNF* gene attribution in pregnant and lactating female mice demonstrated that EBN supplementation improved the newborns' learning and memory performance (82). In the hippocampus area, BDNF expression can increase neurogenesis *via* promoting mitochondrial biogenesis and neuronal plasticity (50, 82).

The expression of genes resulting from dietary sialic acid supplementation has a profound influence on the brain processes such as cell adhesion and signal transduction toward brain cognitive development. It has been reported that sialic acid in EBN supplementation raises brain gene expression associated with improved cognitive performance in the Y maze in both the generations of animals (82). However, it remains unclear whether or not EBN supplementation affects brain gene expression, as the amount of sialic acid in different EBN sources varies. EBN-derived sialic acid exhibited improved cognitive impairment in mice treated at various dosages. Pheochromocytoma and neuroblastoma cells were shown to grow more quickly when EBN was added to their culture (66). Researchers found a link between brain growth and function and sialic acid content in the blood (66). EBN was found to improve memory and learning in Wistar rats exposed to LPS-induced neuroinflammation with sialic acid's anti-inflammatory effects (33).

Menopause causes cognitive dysfunction due to impaired neuronal plasticity in the hippocampus. EBN could be beneficial in the treatment of menopause-related cognitive impairment. Menopause cognitive dysfunction can be alleviated utilizing EBN as a natural supplement, according to Zhiping et al. (83) As a result of this study, researchers discovered that estrogen shortage and downregulation of genes linked to neurodegeneration in the hippocampus and frontal cortex were reduced by EBN. The advanced glycation end products linked with estrogen deprivation were considerably reduced by EBN. EBN also boosts antioxidant enzyme activity to reduce oxidative stress in the hippocampal and frontal cortex (83). The study's findings are in line with those of another study conducted in 2017, which found that administering EBN to ovariectomized rats improved their cognitive abilities in the hippocampus. Neuronal plasticity in the hippocampus, which is linked to cognitive abilities, could be improved by increasing EBN's activity in the brain's Silent Information Regulator 1 *(SIRT1)* gene (69). In addition, EBN is a less harmful therapy option than estrogen. The ovariectomized rat's kidney and liver may be adversely affected by estrogen therapy, despite improving cognitive abilities (69). These findings suggested that EBN may serve as an alternative treatment to ameliorate neurodegenerative diseases in menopause.

Cognitive decline may be caused by a decrease in cerebral blood flow (CBF), which may lead to a chain reaction of inflammation and oxidative stress. Recently, the medical idea has emphasized the significance of natural antioxidant products as a nutritious compound in preserving the brain from physiological changes that cause aging or any neurological illness. Bilateral occlusion of the common carotids (2VO) was used to produce CBF decline in rats, which mimics human aging brain CBF decline (34, 87–89). Ismaeil et al. (34) investigated the neuroprotective effects of EBN on 2VO rat animal model. Neuronal damage and higher oxidative stress were found in the untreated group after long-term carotid artery obstruction. A greater number of viable neuronal cells in the CA1 hippocampal area in the 2VO treated groups revealed an improvement in degenerative alterations of neuronal cells. It has been shown that EBNs antioxidant and anti-inflammatory qualities may have the ability to improve cognitive processes, as demonstrated by its pharmaceutical intervention. To halt the progression of AD, it may be beneficial to consume foods having therapeutic properties. In view of the fact that EBN has long been eaten for medicinal and health reasons, our findings suggest that it may be able to postpone the onset of Alzheimer's-related dementia when taken early in life. As a supplement, it can help to prevent the aging of neurons.

## Current Challenges and Future Perspectives

Despite a lack of scientific study on the therapeutic benefits of EBN in the past, numerous scientific publications have been published on this issue in recent decades. Several studies have demonstrated and summarized these effects, including the notion that EBN is a neuroprotective antioxidant with other health benefits (33, 75). Study must be conducted to fully comprehend the underlying fundamental problems, particularly the molecular and biochemical mechanisms through which EBN acts as a neuroprotective agent. It is required to isolate the individual components that contribute to the neuroprotective antioxidant effect. Furthermore, evidence of the association between EBN doses and its biological activities is urgently needed. Thus, elucidating the molecular mechanisms by which the EBN component exerts its biological effects *in-vivo* and *in-vitro studies* would be a huge achievement. In addition, it would be advantageous to ascribe particular biological functions to certain components of EBN study and then isolate and purify them. The findings and recommendations will provide the strength of evidence and the recommended intake of EBN.

It is plausible to conclude, based on the recent scientific updates, that the composition of EBNs obtained from diverse sources and regions varies. As a result, standardizing the composition of EBNs and developing a standard operating procedure would help to ensure a stable and consistent output. Additional study examining the technique used in this study, as well as the complexity and diversity of the location sources, is necessary to justify the observed variance. If a sample is obtained from a market, a dealer, or a retail establishment, it must be classified as processed due to the high probability of adulteration. Bleaching is a frequent method of adulteration since it conceals the bird feathers. Others include the use of fortified substances such as egg white, jelly, seaweed, or even hog skin to promote weight growth (90). These will surely modify the composition of EBN, thereby affecting the experimental results.

For generations, EBN has been used as a folk remedy for several ailments but has never been utilized as a pharmaceutical to cure or treat the sickness. This is because there has been a shortage of studies on the formulation and appropriate dose of this unique animal-derived bioproduct. To the best of our knowledge, there has been no report of fractionation or separation of a single component from EBN material, meaning that no single component has been shown to be therapeutic. As of now, only *in*-*vitro* and *in*-*vivo* tests using the whole EBN extract have been performed, with no further characterization on its specific constituent. Thus, EBN may only be regarded as food or, at best, a functional food due to a lack of scientific proof and reports.

Although there is evidence that EBN can play a role to prevent diseases, the safety issue is paramount. Many health instances have demonstrated a rise in allergic responses due to EBN use. In Japan, allergic symptoms such as skin rash, nasal obstruction, and facial edema have been reported within 5 min after ingesting an EBN-containing dessert. Allergic reactions vary in intensity and severe cases might end in death (91). A similar instance was described by the National University of Singapore, in which EBN produced food-induced anaphylaxis in children. Anaphylaxis can occur in the presence of putative allergens and when the immune system's immunoglobulin E-mediated mechanism is not properly regulated (91). As a result, it is critical to assess a person's sensitivity or susceptibility to EBN protein allergies before consumption using a skin prick test. These studies established EBN as a possible allergen. The study, which originated at Singapore's prestigious National University, raises grave concerns. However, because the test samples were obtained from the market, it is possible that they were tampered with along the way by the bird's premises handler or producer to increase profit. The term “egg white-like” protein is a reasonable description, as the EBN processor would generally add egg white to the surface of EBN to give it a good shine and, hence, attract a higher price (92). A decent understanding and knowledge of consumer market norms and behaviors will ensure the use of a representative sample in study, resulting in a more reliable conclusion.

Although EBN is a promising material, study on it is limited. Nonetheless, only a few study have shown the impact of EBN on cognitive function and these studies have been carried out with limited emphasis on *in-vivo* studies. Thus, more studies on whether supplementation improves cognitive function, including behavior studies, are warranted. Furthermore, there is a lack of standardization in terms of the EBN composition as it can be concluded that the composition of EBN significantly varies from one location to another location. These would deviate the results of experiments. In addition, the efficacy dose of the EBN also needs to be studied to attenuate oxidative stress and neuroinflammation. To better understand the EBNs anti-inflammatory properties, this study demands further inflammatory markers to be tested. Finally, the hippocampus and cerebral cortex of the animal models should be examined in future studies to determine gene and protein expression.

## Conclusion

Edible bird's nest is a rich source of amino acids and carbohydrates with reported health-promoting ingredients. Owing to its health benefits, EBN has now been developed into various food products, including beverages and food additives. However, study on the development of EBN as a potential supplement of neurodegenerative diseases is still scant. Therefore, this exhaustive overview of EBN should promote further study, especially on proteomic and genomic area to fully understand its compositions and functions. Nutritional and pharmacological properties attributed to EBN should be supported by extensive sound and reliable study, especially on the safety and efficacy of EBN. In summary, EBN, its extract, and product have a great potential for future development as a cognitive enhancer in the treatment of neurodegerative diseases.

## Author Contributions

All authors listed have made a substantial, direct, and intellectual contribution to the work and approved it for publication.

## Conflict of Interest

The authors declare that the research was conducted in the absence of any commercial or financial relationships that could be construed as a potential conflict of interest.

## Publisher's Note

All claims expressed in this article are solely those of the authors and do not necessarily represent those of their affiliated organizations, or those of the publisher, the editors and the reviewers. Any product that may be evaluated in this article, or claim that may be made by its manufacturer, is not guaranteed or endorsed by the publisher.
